# Comparative Study of Structural Changes of Polylactide and Poly(ethylene terephthalate) in the Presence of *Trichoderma viride*

**DOI:** 10.3390/ijms22073491

**Published:** 2021-03-28

**Authors:** Grażyna B. Dąbrowska, Zuzanna Garstecka, Ewa Olewnik-Kruszkowska, Grażyna Szczepańska, Maciej Ostrowski, Agnieszka Mierek-Adamska

**Affiliations:** 1 Department of Genetics, Faculty of Biological and Veterinary Sciences, Nicolaus Copernicus University in Toruń, Lwowska 1, 87-100 Toruń, Poland; browsk@umk.pl (G.B.D.); znajewsk@doktorant.umk.pl (Z.G.); 2 Department of Physical Chemistry and Physicochemistry of Polymers, Faculty of Chemistry, Nicolaus Copernicus University in Toruń, Gagarina 7, 87-100 Toruń, Poland; olewnik@umk.pl; 3 Laboratory for Instrumental Analysis, Faculty of Chemistry, Nicolaus Copernicus University in Toruń, Gagarina 7, 87-100 Toruń, Poland; gina@umk.pl; 4 Department of Biochemistry, Faculty of Biological and Veterinary Sciences, Nicolaus Copernicus University in Toruń, Lwowska 1, 87-100 Toruń, Poland; maciejost@umk.pl

**Keywords:** polylactide, poly(ethylene terephthalate), *Trichoderma viride*, hydrophobin, hydrophobin film, bioremediation

## Abstract

Plastic pollution is one of the crucial global challenges nowadays, and biodegradation is a promising approach to manage plastic waste in an environment-friendly and cost-effective way. In this study we identified the strain of fungus *Trichoderma viride* GZ1, which was characterized by particularly high pectinolytic activity. Using differential scanning calorimetry, Fourier-transform infrared spectroscopy techniques, and viscosity measurements we showed that three-month incubation of polylactide and polyethylene terephthalate in the presence of the fungus lead to significant changes of the surface of polylactide. Further, to gain insight into molecular mechanisms underneath the biodegradation process, western blot hybridization was used to show that in the presence of poly(ethylene terephthalate) (PET) in laboratory conditions the fungus produced hydrophobin proteins. The mycelium adhered to the plastic surface, which was confirmed by scanning electron microscopy, possibly due to the presence of hydrophobins. Further, using atomic force microscopy we demonstrated for the first time the formation of hydrophobin film on the surface of aliphatic polylactide (PLA) and PET by *T. viride* GZ1. This is the first stage of research that will be continued under environmental conditions, potentially leading to a practical application.

## 1. Introduction

The undeniable advantages of polymers, including their durability, light weight, cheapness, and excellent mechanical properties, mean that plastics are used for innumerable purposes resulting in enormous amounts of plastic waste all over the world [1,2]. Plastic pollution is one the most important global threats for the environment and for public health. The increased demand for polymers means that storing and managing plastic waste is a growing problem worldwide. Thus, in recent years an increasing interest in polymers susceptible to degradation that can be accelerated by biological factors, e.g., soil fungi, has been observed [3,4]. 

A greater interest in aliphatic polylactide (PLA) as a packaging material is not only due the functional properties of lactic-acid-based polymers but also due to their degradability in the natural environment. The biological degradation of PLA depends on the chemical structure of the polymer, the share of crystalline phase, and the hydrophilicity [5,6]. However, despite PLA being a biodegradable polymer, in soil the initiation of the degradation process takes a long time and depends on the temperature [7,8]. The other widely used polymer is the aromatic polyester poly(ethylene terephthalate) (PET), the main components of which are terephthalic acid and ethylene glycol [9]. The degradation time of PET in the environment has been estimated in the range of between 16 and 48 years [10]. PET is often compared to PLA in terms of its tensile strength, modulus of elasticity, impact resistance, and barrier properties, and both are widely used [11]. PET is widely used for the production of bottles, food wrappers, microwave trays, and construction pipes [12], whereas PLA is used as a food packing material—however, recently the potential of this polymer for biomedical application (e.g., suture threads, skin draft, and nanoparticles for drug delivery) has been widely discussed [13]. 

Currently, polymer waste is managed mainly by landfilling, incineration, and recycling [14]. As an alternative to traditional methods, biodegradation has emerged. It is usually a more cost-effective and more efficient process that does not produce the secondary pollution associated with traditional methods [15,16]. The main role in biodegradation is played by microorganisms, including fungi, that can biodegrade a range of plastic types, e.g., fungi of the genera *Aspergillus*, *Fusarium*, *Chaetomium*, *Peacilomyces*, *Mucor*, *Cryptococcus*, *Rhizopus*, *Penicillium* [17,18], *Pseudozyma japonica* [19], *Clitocybe* sp., and *Laccaria laccata* [20]. Fungi of the genera *Aspergillus*, *Trichoderma*, *Fusarium,* and *Alternaria* have the ability to degrade polyurethane in soil [21], whereas the fungi *Aspergillus ustus*, *Aspergillus sydowii*, and *Aspergillus fumigatus* are involved in the biodegradation of PLA [5]. Cutinase from *Fusarium solani pisi* shows hydrolytic activity towards PET [22] and has been the most extensively characterized as a PET degrading enzyme. All known PET hydrolases belong to the cutinase group [9]. Yoshida et al. [23] identified a PETase from the bacterium *Ideonella sakaiensis* that prefers PET to aliphatic esters, compared to cutinases from other organisms.

*Trichoderma* spp. fungi, that belong to type *Ascomycota*, occur in soils at various latitudes. They grow very quickly, sporulate abundantly, and can produce several hydrolytic enzymes. The presence of these fungi in soil depends on abiotic factors such as soil pH, humidity, and temperature. Species belonging to *Trichoderma* are usually the most abundant fungi species in almost all soils in temperate and tropical climates. It is estimated that every gram of soil contains an average of 10^1^ to 10^3^ *Trichoderma* spores that are ready to develop [24]. They are also root endophytes of many plant species and it was shown that those fungi were able to promote plant growth and development and to induce plant resistance systems [24,25]. In our previous research it was demonstrated that *Trichoderma viride* is effective in degrading polycaprolactone (PCL) [16]. Moreover, it was shown that *T. viride* is able to grow on PLA, PET, PCL, and polyethylene (PE) films [3]. 

An important factor facilitating the biodegradation of plastics by filamentous fungi of types *Ascomycota* and *Basidiomycota* is their ability to produce small (7–15 kDa), extracellular, cysteine-rich proteins—hydrophobins. These proteins perform numerous functions during growth and development of fungi, such as to mediate interactions between the fungus and the environment through allowing attachment of hyphae to hydrophobic surfaces [26,27,28]. Characteristic features of these proteins are their ability to self-assemble [29,30], the ability to cover various surfaces and reduce surface tension, the ability to spontaneously form into amphipathic monolayers, and the ability to constitute a hydrophobic film between hydrophobic and hydrophilic environment [28,31]. The hydrophobic film can change the nature of various surfaces from hydrophobic to hydrophilic and vice versa [26,32]. Hydrophobins give mycelial cells hydrophobic properties that increase the ability of fungi to adhere to various surfaces, including plant tissues [33]. Moreover, hydrophobins allow fungi to adhere to plastics [34], which seems to be of key importance for biodegradation processes [12].

There are two classes of hydrophobins. Class I includes proteins that are very poorly soluble in aqueous solutions and dissociate only in concentrated acids [35,36]. Class I hydrophobins have been identified in *Ascomycota* and *Basidiomycota* fungi. In contrast, class II hydrophobins are readily soluble in the aqueous solutions of organic solvents [37]. To date, proteins belonging to class II have been identified in ascomycetes only [28]. Recent research has indicated the possible existence of a new group of hydrophobins sharing properties with both class I and class II hydrophobins. These proteins have been described in fungi belonging to the genera *Trichoderma* and *Aspergillus*. They differ in the arrangement of their cysteine residues and their hydropathy patterns from class I and class II hydrophobins, and also from each other. It has been speculated that hydrophobins are much more diversified than it was initially assumed, and thus a more complex classification system would be needed [38,39]. Moreover, fungi produce other surface-active proteins, such as cerato-platanin, which are significantly different in structure from hydrophobins [40]. 

The potential of hydrophobins in the degradation process of polymers has been already demonstrated. Hydrophobins have been shown to allow fungi to adhere to the hydrophobic surface of PET [34]. Moreover, the fusion of cutinase [41] and PET hydrolase [42] with hydrophobins significantly increased the level of PET degradation.

The aim of this study was to identify a microorganism common in the environment and to analyze the reaction of the microorganism to the presence of two polymers—the biodegradable polylactide (PLA) and the poorly biodegradable poly(ethylene terephthalate) (PET). We identified the saprophytic fungi *Trichoderma viride* strain GZ1 as showing mycoparasitic ability. We analyzed the identified strain in order to: (i) establish whether *T. viride* GZ1 produced hydrophobin proteins that enable the attachment of hyphae to the surface of polymer materials, (ii) determine whether hydrophobin film is present on the surface of PLA and PET by atomic force microscopy, and (iii) monitor the changes in the structure and chemical properties of PLA and PET caused by the presence of fungus using scanning electron microscopy, differential scanning calorimetry, viscosity measurements, and Fourier-transform infrared spectroscopy. The long-term goal of our research is to develop a biopreparation containing *T. viride* GZ1 spores that could be used in municipal landfills to accelerate the degradation of plastic waste.

## 2. Results

### 2.1. Identification and Characterization of T. viride GZ1 Metabolic Activity

The ITS (internal transcribed spacer) sequence obtained by PCR reaction (deposited in GeneBank under accession number MT584875) showed the highest similarity to ITS sequences from *T. viride* (e.g., isolate OTU220 acc. no. GU934667.1 and isolate F120 acc. no. KJ482541.1) with over 99% matching identities. This molecular identification confirmed that the strain used in this study belonged to *T. viride*. 

The hydrolytic activity (W_act_) of the *T. viride* GZ1 strain was analyzed. It was shown that this strain possessed pectinolytic (0.0917 ± 0.0041), lipolytic (0.0599 ± 0.0019), cellulolytic (0.0591 ± 0.0020), amylolytic (0.0524 ± 0.015), and proteolytic (0.0458 ± 0.0030) activities. The fungus was found to be very effective, especially at pectin degradation, and pectinolytic activity was twice as high as proteolytic activity, which was the lowest hydrolytic activity in this strain. 

### 2.2. Adhesion and Growth of T. viride GZ1 on Polymer Material

**Figure 1:**
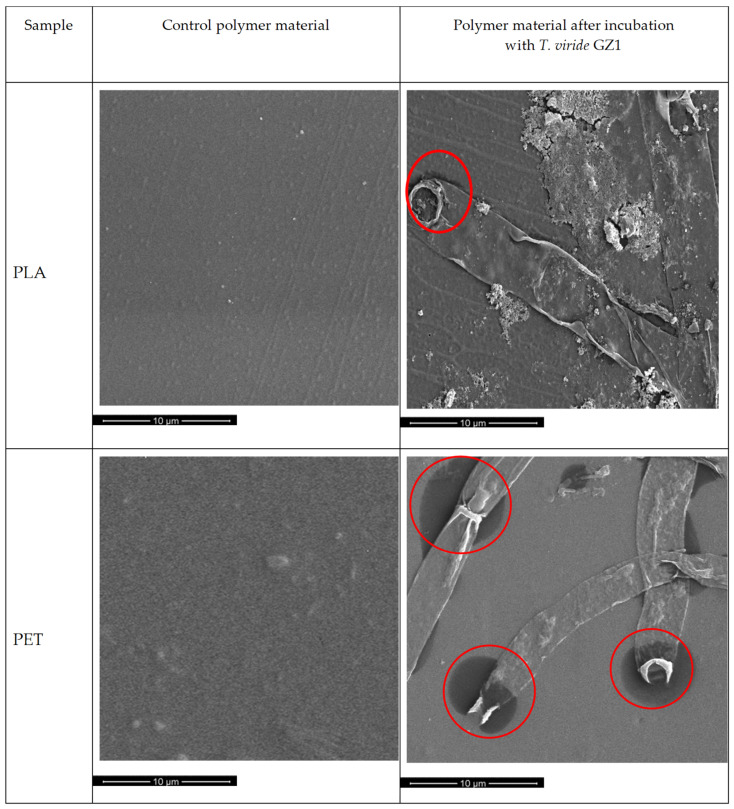
SEM analysis (magnification 10,000×) of the polymer materials after 3-month incubation in liquid medium: the left-hand side shows polymers incubated without fungus (control), and the right-hand side shows polymers incubated with *T. viride* GZ1. Red circles indicate spots where the release of the cytoplasmic content of hyphae takes place.

In order to evaluate, in detail, changes caused by *T. viride* GZ1 on the surface of PLA and PET, polymers before and after exposition to the *T. viride* were analyzed by SEM (Figure 1). Before incubation with the fungus, the surface of PLA, as well as PET, samples were smooth and without cracks (Figure 1, left-hand side). After 3-month incubation with fungi it was clearly seen that the surface of PLA, as well as PET, was covered with hyphae of *T. viride* GZ1. As can be observed, some developing fungi hyphae managed to adhere to the surface of the films very tightly and were still present on the surface after rinsing. Interestingly, some darker (more intense color) spots were visible on the surface of both polymers; however, they were particularly noticeable on PET films (Figure 1). Those darker spots might indicate the release of the cytoplasmic content of hyphae. 

### 2.3. Analysis of PET and PLA Biodegradation by T. viride GZ1

#### 2.3.1. Thermal Properties

**Figure 2:**
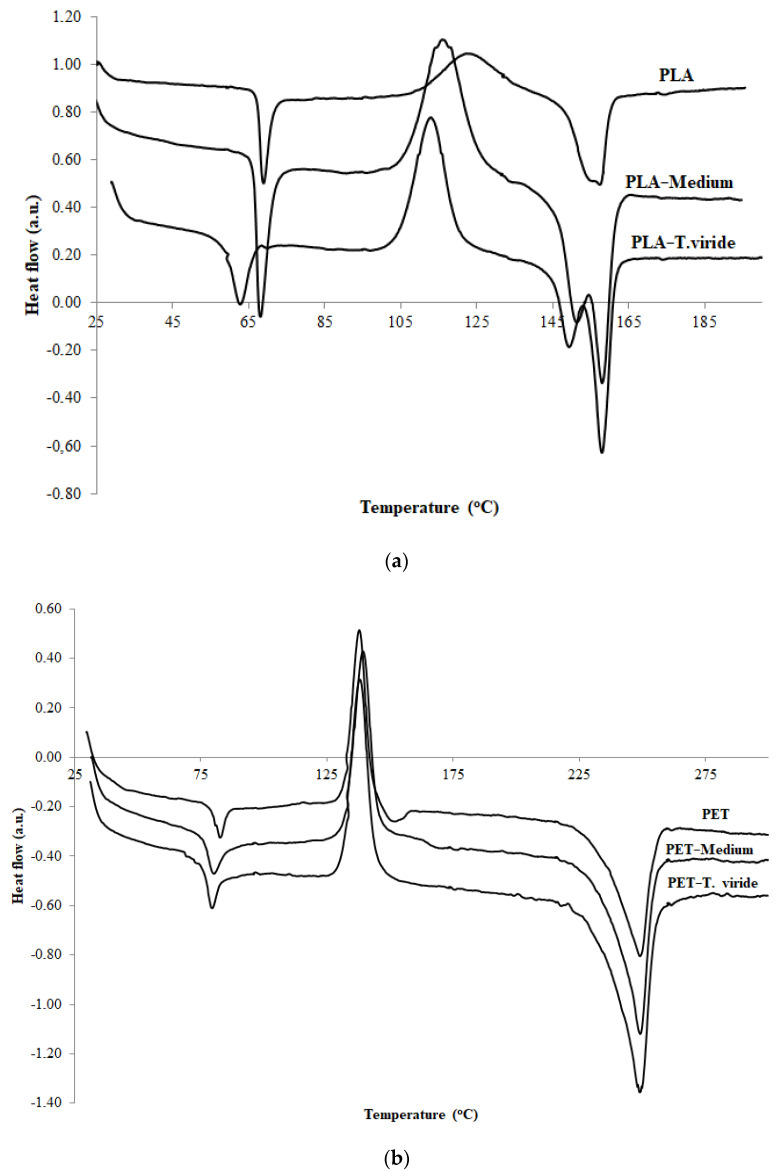
Differential scanning calorimetry (DSC) thermograms of (**a**) aliphatic polylactide (PLA) and (**b**) poly(ethylene terephthalate) (PET), before treatment (PLA/PET), after 3-month incubation in clean medium (PLA/PET−medium), and after 3-month incubation in medium containing *T. viride* GZ1 (PLA/PET−T. viride).

During the contact of the fungus with PLA and PET, the thermal properties of the polymer materials changed, which was related to the decrease in molecular weight, and to the decomposition of the amorphous phase of degraded materials, as well as to the formation of degradation products. The glass transition temperature (T_g_) of PLA (Figure 2a) and PET (Figure 2b) films decreased in comparison with films before the incubation with the fungus, or with films incubated in medium only. However, the differences between the T_g_ value for the polymers before incubation and the T_g_ value for the polymers after incubation with fungus was significantly different for both polymers: for PET this difference equaled about 3 °C while for PLA it exceeded 7 °C. The same effect was noticed in the case of cold crystallization temperature (T_c_). The value of T_c_ in the case of PET film slightly increased for material incubated with the fungus, which clearly suggests that shorter polymer chains diminished during degradation (Figure 2b).

The values of all studied thermal parameters of PLA and PET films before treatment, after incubation in medium, and after *T. viride* GZ1 exposure are shown in Table 1. It can be clearly seen that in the case of both studied polymers, after incubation in medium as well as after exposure to fungi, the values of enthalpy of crystallization (∆H_c_) and of melting enthalpy (∆H_m_) were increased (Table 1). 

#### 2.3.2. Fourier-Transform Infrared Spectroscopy (FTIR)

**Figure 3:**
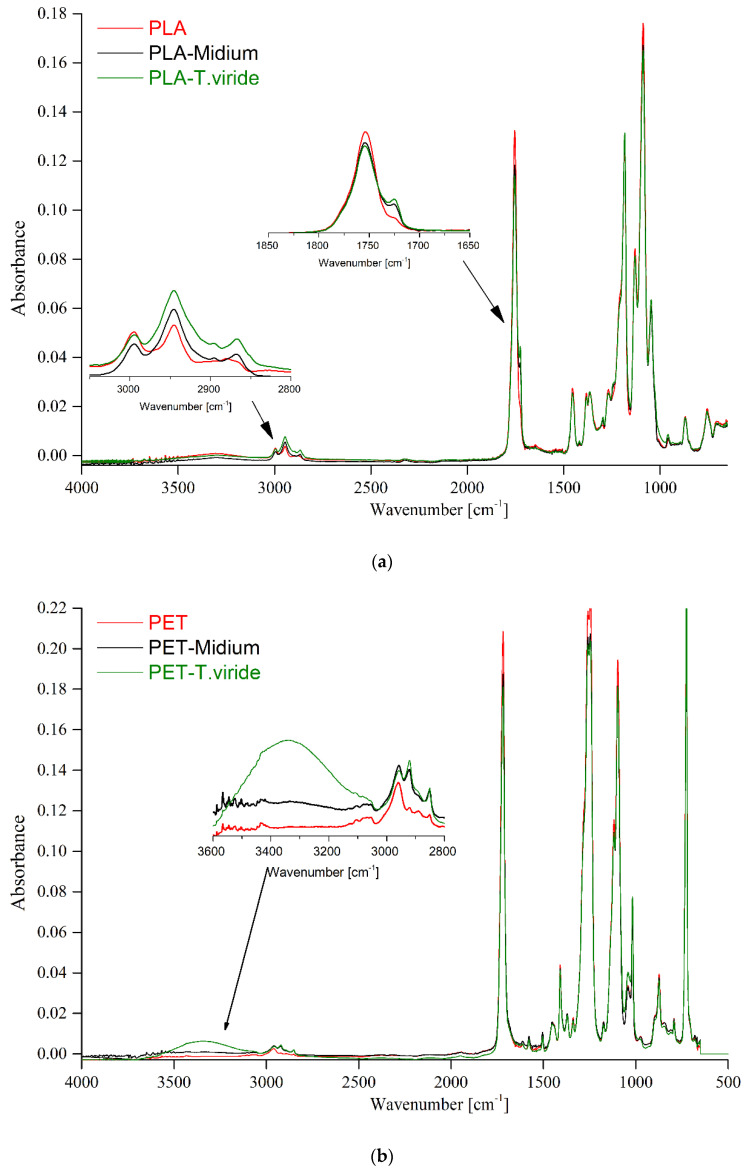
Infrared spectra of (**a**) PLA, and (**b**) PET, before treatment (red line), after 3-month incubation in medium (black line), and after 3-month incubation in medium-*T. viride* GZ1 (green line).

In order to further analyze the changes in the structure of the studied polymers after incubation with fungi, the FTIR-ATR (attenuated total reflection) analyses were performed (Figure 3a,b). The molecular structure of neat PLA was described previously by our group [43], and the main spectral absorption of the functional groups present in PET was described by Mecozzi and Nisini [44]. After the incubation of PLA in the medium and after the incubation of PLA with fungi, a new absorption band at 1724 cm^−1^ was observed (Figure 3a) that corresponds to the carboxylic acid, which emerged possibly as a result of the biodegradation of PLA. Moreover, the increase in the intensity of the absorption bands at 2995 and 2944 cm^−1^, assigned to symmetrical and asymmetrical stretching vibration of CH_3_ and CH_2_ groups, can be observed [45]. It can be clearly seen that the intensities of those emerging absorption bands were higher for PLA films incubated with fungi than that for PLA films incubated in medium only. The infrared spectra of PET films, PET films incubated in medium, and PET films incubated with the fungus (Figure 3b) have shown that the most significant changes in absorbance were present in the range between 3000 and 3500 cm^−1^. Within the mentioned range the band was clearly associated with the -OH groups, which may have been derived from the degradation products and hydrogen bonds. However, it is possible that this band was due to the water adsorbed on the modified surface of PET. 

Taking into account that the samples were analyzed by FTIR-ATR technique, it is reasonable to claim that the IR spectra clearly show that the exposure to fungi significantly changed the molecular structure of not only biodegradable PLA films but also of PET films. 

### 2.4. Determination of Molecular Weight of Polymers Incubacted with the Fungus

Based on the flow time of the particular polymer solution and a pure solvent, the relative viscosity (*η_r_*) as well as specific viscosity (*η_sp_*) were calculated according to one of the following Equations (1) and (2):

(1) ηr=tt0

(2) ηsp=ηr−1=tt0−1

**Figure 4:**
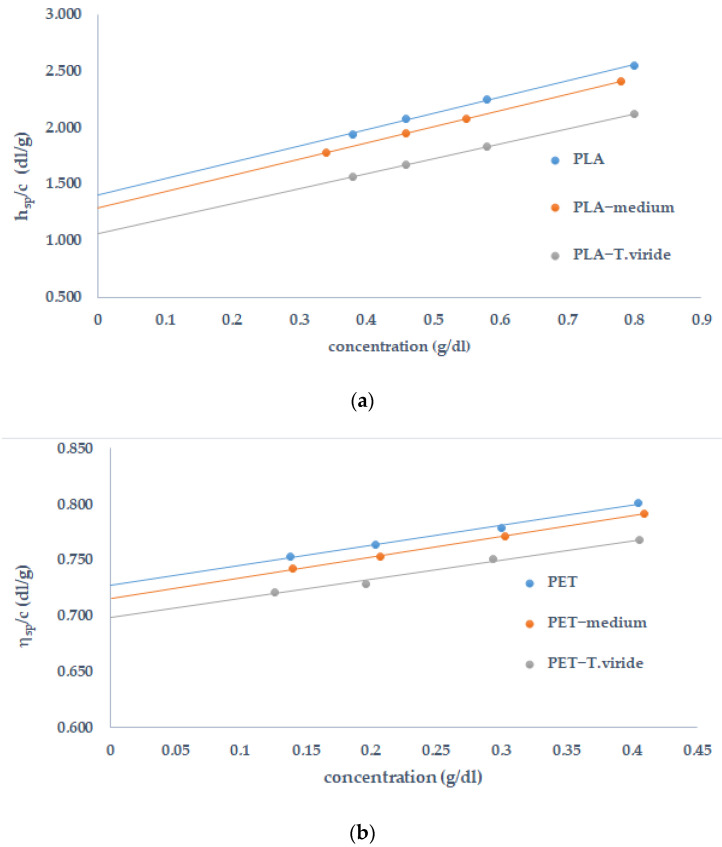
Reduced viscosity *η_sp_/c* versus (**a**) PLA and (**b**) PET concentration, before and after the incubation of the polymer in medium (PLA/PET−medium) or in fungal culture (PLA/PET−T. viride).

In the first stage, the specific viscosity was used to determine intrinsic viscosity ([η]). For this reason, the changes of *η_sp_/c* in the function of a polymer’s concentration, before and after polymer degradation, were analyzed (Figure 4). It should be stressed that intrinsic viscosity describes the molecular density of the studied polymer.

Obtained values of intrinsic viscosity ([η]) allowed us to calculate changes in the number average molecular weight of the polymer, according to the Mark–Houwink Equation (3):

(3) $$\left[\eta \right]=\kappa {\bar{M_{v}}}^{\alpha }$$

where κ and α are the constants characteristics of each polymer–solvent system.

Based on the formulas described in the literature [46,47], the number average molecular weight was determined using the following formulas: (4) for PLA and (5) for PET, respectively.

(4) $$\left[\eta \right]=3.25\;\times 10^{-4}{\bar{M_{n}}}^{0.77}$$

(5) $$\left[\eta \right]=3.72\;\times 10^{-4}{\bar{M_{n}}}^{0.73}$$

The values of intrinsic viscosity and number average molecular weight are presented in Table 2.

It can be clearly seen that in the case of PLA the degradation had started in the medium, however, a more significant decrease in the intrinsic viscosity as well as in molecular mass was observed after exposure to *T. viride*. The changes in molecular mass justify the conclusion that *T. viride*. significantly influences the decomposition of the polylactide. The measurements of intrinsic viscosity and the values of molecular weight before and after PET exposure to different mediums provide detailed information regarding the degradative processes. The effect of the PET exposure to the medium as well as *T. viride* indicates that there is no significant difference in viscosity when compared with the film before incubation with the fungus. However, the lowest value of M_n_ was observed after the incubation of PET caused with *T. viride*. 

### 2.5. Hydrophobins

#### 2.5.1. Detection of Hydrophobins in Fungal Culture

**Figure 5:**
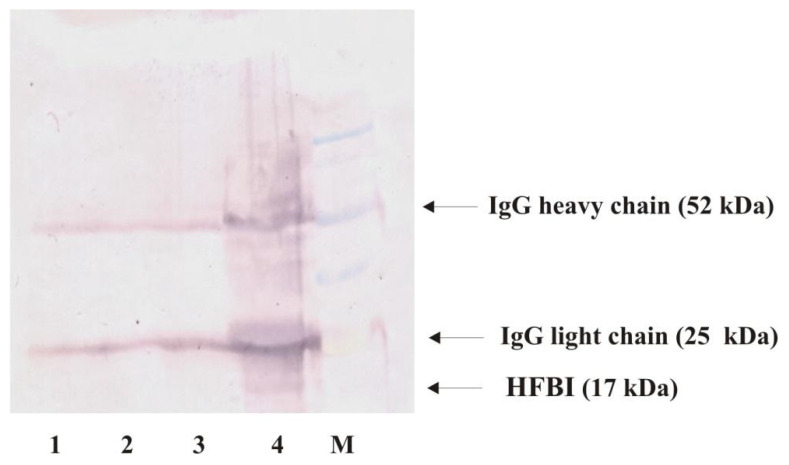
Western blot analysis of hydrophobin. Proteins were immunoprecipitated using anti-HFBI antibody, and resulting pellets were washed and subjected to SDS-PAGE/Western blot analysis. Lane 1: liquid medium, lane 2: *T. viride* GZ1 culture, lane 3: liquid medium with PET, lane 4: *T. viride* GZ1 culture with PET, and M: molecular mass standard (BluEasy Prestained Protein Marker, Nippon Genetics, Dürren, Germany). All analyzed media/cultures were incubated for 3 months. Arrows indicate IgG heavy (52 kDa) and light (25 kDa) chains and protein that corresponds to HFBI (17 kDa).

Direct Western blot analysis of the 3-month-old liquid fungal culture did not show any protein band, probably due to the low level of hydrophobin proteins excreted to the culture [48]. Therefore, we decided to use Protein A immunoprecipitation prior to Western blot analysis. As shown in Figure 5, a single protein band that corresponded to hydrophobin (HFBI; molecular mass of 17 kDa) was observed only in the *T. viride* GZ1 culture containing PET. Two additional protein bands (52 and 25 kDa) corresponded to the heavy and light chains of IgG, respectively.

#### 2.5.2. Detection of Hydrophobin Film

**Figure 6:**
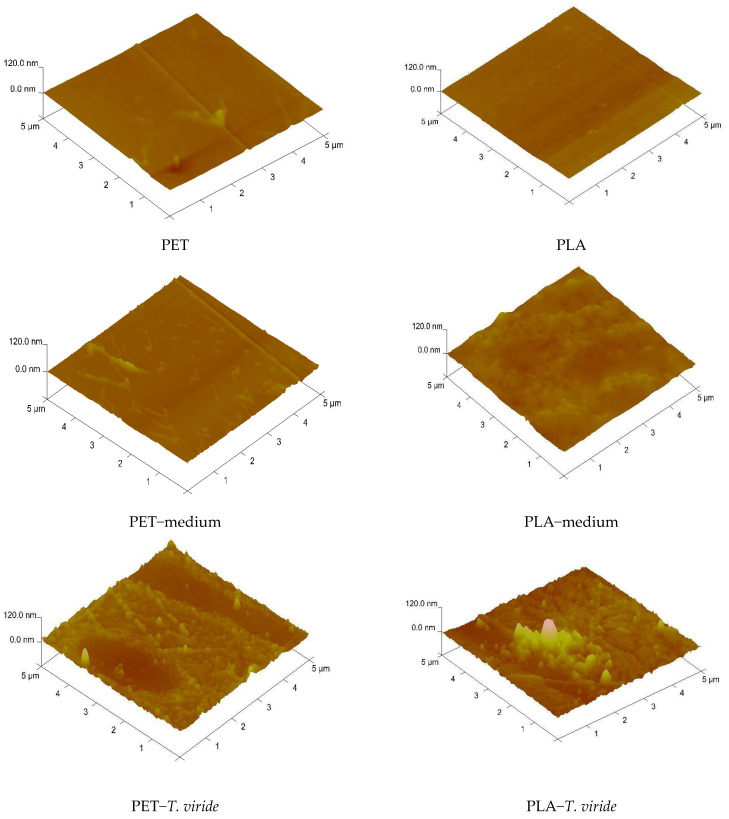
Atomic force microscopy (AFM) images of PET (left-hand side) and PLA (right-hand side) before treatment, after 3-month incubation in medium, and after 3-month incubation in medium-*T. viride* GZ1.

Atomic force microscopy (AFM) was used to study the changes of the PET and PLA surfaces, and the possibility of the presence of hydrophobin film on the surface of the polymers after incubation with fungi. Figure 6 presents AFM images of the representative surface topology of the analyzed polymers before treatment, after 3-month incubation in medium, and after 3-month incubation with *T. viride* GZ1. Table 3 presents the values of the different roughness parameters (*R_a_, R_q_, R_max_*) for corresponding samples which were calculated based on three-dimensional images.

The obtained results clearly show that the surfaces of PET and PLA films before contact with fungi did not display significant alterations in topography. Both homopolymers exhibited a uniform surface, which was also reflected by the values of *R_a_* and *R_q_*, that did not exceed 4 nm (Table 3). However, after incubation of the polymers in medium only and in *T. viride* GZ1 culture, *R_a_* and *R_q_* values changed significantly. It is clearly visible that the surface roughness of both polymers increased. In the case of the PET sample, the *R_q_* value was 4.19 nm after incubation in medium only, while after incubation in the presence of *T. viride* GZ1 the *R_q_* parameter was almost 10 nm. However, the *R_a_* value, which is the arithmetic average of the absolute values of the profile heights over the evaluation length, was the highest for the PET-*T. viride* sample. The increase in roughness parameters was also observed in the case of PLA films, and they were significantly higher after treatment of PLA with *T. viride* GZ1. Moreover, it can be seen that the surface of polymers incubated with the fungi were covered by rodlets of hydrophobins that were present on the surface even after the washing of films prior to AFM analysis (Figure 6).

## 3. Discussion

Growing consumption of goods and services forces the increasing production and usage of polymer plastics. Disposal of these in landfills poses a serious threat to the environment and all living organisms. Therefore, there is an urgent need to develop expeditious and environment-friendly approaches to accelerate the degradation of plastic waste. Bioremediation is one such effort, and includes usage of microorganisms, mostly bacteria and fungi, to degrade a range of polymer materials. However, depending on the chemical structure, the susceptibility of polymers to biodegradation is varied. Therefore, our research has explored mechanisms that might effectively accelerate the biodegradation of two common polymers—PLA and PET. PET is considered to be relatively resistant to biodegradation, whereas PLA is listed among biodegradable polymers but is more resistant to microbial attack in the environment than synthetic polyesters [49,50].

### 3.1. Identification and Metabolic Activity of T. viride GZ1

In our study we used fungi belonging to *Trichoderma,* that are some of the most frequently occurring microorganisms in soil all over the world. Studies on *Trichoderma* diversity and speciation have determined that ribosomal RNA regions ITS1, 5.8S, and ITS2 are among the most informative sequences, in terms of taxonomy and phylogeny [50]. Therefore, for molecular identification of the species of fungi used in this study, the genomic region including ITS1, 5.8S rRNA gene, and ITS2 was amplified and sequenced. This analysis confirmed that this fungus belonged to *T. viride*.

It has been already reported that enzymes including cutinases, laccases, lipases, and enzymes involved in lignin metabolism are involved in the degradation of polymers [51,52,53,54,55]. *T. viride* GZ1 has been shown to produce pectinases, lipases, cellulases, and proteases at a high level, which might suggest that this strain has potential for biodegradation of polymers. There are several pieces of evidence in the literature that those enzymes are involved in polymer biodegradation. Research by Carniel et al. [56] showed that lipases and cutinases produced by *Candida antarctica* and *Humicola insolens*, respectively, caused structural changes of PET plastic. Moreover, Janczak et al. [20] showed that the fungi *Laccaria laccatta* and *Clitocybe* sp., which show cellulolytic activity, and also pectinolytic activity in case of *Clitocybe* sp., accelerated degradational changes of PLA and PET. PLA can be hydrolyzed by lipase from *Rhizopus delemar* and proteinase K from *Tritirachium album*, and also by polyester polyurethane depolymerase from *Comamonas acidovorans* [57,58]. A study by Nimchua et al. [59] showed that 22 out of 115 fungal isolates obtained from the surfaces of plants and rhizosphere soil had the capacity to modify PET films. A bacterium, *Ideonella sakaiensis*, was isolated from a PET-contaminated environmental sample, and it was shown that it not only degraded PET, but also assimilated the monomers [23]. The hydrolysis of PET by the fungi *Fusarium oxysporum* LCH1 and *Fusarium solani* was demonstrated. It was also shown that hydrolases from those two fungal species differed in efficiency, indicating that species belonging to the same genus may have varied potential for polymer biodegradation [60]. The *T. viride* GZ1 strain used in this study was isolated from the surface of the fungus *Cerioporous squamosus*, and the identification of the hydrolytic activities of this strain confirmed not only the potential of this strain for biodegradation but also its ability for mycoparasitism. High pectinolytic and cellulolytic activity of the strain used in this study indicates that, other than biodegradation, practical usages are possible. *Trichoderma* sp. possessing high cellulolytic and pectinolytic activity was able to accelerate compost decomposition [61]. Mutschlechner et al. [62] used the *T. viride* strain with high cellulolytic activity to increase biogas production. This suggests that fungi belonging to *Trichoderma* have great potential for varied practical applications within waste management.

### 3.2. Changes in the Structure and Thermal Properties of PET and PLA after Incubation with the Fungus

In general, the biodegradation of polymer materials requires the activity of several different microorganisms [63]. The level of biodegradation is affected by polymer chemical structure and the microorganisms present in a certain environment. Crystallinity, molecular weight, hydrophobicity, the presence of functional groups, and the use of additives and plasticizers in the fabrication process affect the susceptibility to biodegradation. For example, an increase in crystallinity and molecular weight negatively affects biodegradation. On the other hand, pre-treatments of polymers, such as irradiation, increase the susceptibility of the polymer to biodegradation [64]. The durability of plastic is the main obstacle to its degradation in the environment [65,66]. Our previous studies have shown that *Trichoderma* sp. can accelerate the degradation of polycaprolactone [16]. Kannahi and Thamizhmarai [17] identified approximately 40 different fungi that degraded polymers, including species of the genera *Aspergillus*, *Fusarium*, *Chaetomium*, *Peacilomyces*, *Mucor*, *Cryptococcus*, *Rhizopus*, and *Penicillium*. Furthermore, Urbanek et al. [67] isolated 102 microorganisms capable of degrading PCL plastic, including *Trichoderma sp.* fungi, which showed the greatest ability to biodegrade PCL. In addition, it has also been shown that the intense growth of many fungi on a polymer surface can cause blistering and cracking as the fungi penetrate the material structure [17]. The potential of *T. viride* for polymer biodegradation was noted by Munir et al. [68], who isolated several strains from landfill sites. These strains were shown to have the potential to biodegrade LDPE (low-density polyethylene), which is one of the least degradable plastics used in the industry. Strains of both *T. viride* and *Aspergillus nomius* were able to grow in the presence of plastic, and caused a weight loss of polymer of approximately 5–7% after 45 days of incubation in medium with mineral salts. Furthermore, fungi of the genera *Trichoderma*, *Alternaria*, *Aspergillus*, and *Fusarium* were able to degrade polyurethane in soil [21]. Results from Lipsa et al. [69] showed that in the presence of *T. viride* the degradation of PLA occurred more rapidly.

Our SEM analysis results indicate that changes in the color of the surface of the polymers are the result of the presence of fungus hyphae, since they were primarily seen at the fringe of the mycelium and on the intersections of particular hyphae. These observations lead to the conclusion that the degradation products can potentially be used by the fungus as a source of carbon. Moreover, the observed changes in the structure of both studied materials exposed to medium and to *T. viride* GZ1 indicate that the decomposition process might be initiated by the medium and further enhanced by the fungus. The biodegradation products of PLA films include carboxylic acid, whereas in the case of the PET sample, after the three-month incubation with the fungus, products with an -OH group seemed to be present. Moreover, the changes in thermal parameters of PLA and PET samples incubated with fungus were also observed. The observed decrease in the glass transition temperature (T_g_) was due to altered chain mobility and could be the plasticizing effect of the emerging degradation products [70]. The decrease in T_c_ observed for PLA incubated with fungus indicates that shorter chains are formed during the incubation that crystalize at a lower temperature [71]. It needs to be emphasized that for PLA samples, after incubation, the bimodal peak of melting was observed, which indicates that different crystalline forms of PLA were formed. The peak at the lower temperature corresponds to the α’ form, while the peak at the higher temperature is related to the α form [72]. It is well known that the crystalline forms of PLA are strongly related to molecular weight. Therefore, it may be hypothesized that the two degradation products of different molecular weight were the result of the difference in degradation between the surface and the inner layer of PLA. Moreover, based on the obtained results it is reasonable to claim that shorter chains affected the crystallization process of the studied PLA films. The changes of thermal parameters were more visible for PLA due to the aliphatic structure of polylactide. This finding is consistent with the AFM results, which show that the increase in roughness parameters was higher in the case of PLA than in the case of PET.

The effect of *T. viride* on the structural changes of PLA and PET was further analyzed by viscometric tests, which allowed for the calculation of the changes in intrinsic viscosity and number average molecular weight of the studied polymers. It is well known that a higher value of weight-average molar mass relates to a higher intrinsic viscosity value. This phenomenon is due to polymer/solvent interaction. The obtained results show that polylactide was more susceptible to biodegradation compared to PET, since the changes in intrinsic viscosity and number average molecular weight after incubation with *T. viride* were more significant for PLA than for PET. These results are consistent with the DSC results, which show that the lowest values of thermal parameters were recorded for PLA incubated in fungal culture. The susceptibility of polylactide to biodegradation has been widely described in the literature [73,74,75,76,77]. The obtained results of changes in the number average molecular weight confirm that the biodegradation process of polylactide started in the medium and was enhanced by *T. viride.* After incubation of PET in the medium as well as after exposure to *T. viride*, the changes in the number average molecular weight were not as great as in the case of PLA. However, a slight influence of *T. viride* on molecular mass of PET can be seen. The discussed results are consistent with a study of biodegradation of PET described in the work of Nakkabi et al. [78], where a low level of PET degradation by *Bacillus subtilis* was observed. The structure of the analyzed materials played a crucial role during biodegradation. In the work of Farzi et al. [79] it was proved that the biodegradation of PET in the form of powder was more efficient.

### 3.3. T. viride GZ1 Hydrophobin Proteins and Their Potential in Biodegradation

Biodegradation is defined as the capacity of a microorganism or microbial consortium to use the polymer as a sole source of carbon and energy. Fungi belonging to the genus *Trichoderma* have proven to have good environmental adaptability and an ability to use diverse organic and inorganic compounds as a carbon source [80]. These features make *Trichoderma* sp. fungi promising candidates for biodegradation purposes. Moreover, *Trichoderma* sp. have been shown to have the most numerous class II hydrophobin protein families among *Ascomycota* [27,33]. During fungal growth, hydrophobins are secreted by the hyphae to the surrounding environment. In the conditions of low nutrient content, which was the condition of our experiment (minimal medium), the presence of hydrophobins significantly decreases the surface tension of water [81]. Filamentous fungi in the presence of hydrophobins break the water–air barrier and colonize new surfaces, including the surfaces of polymers present in the medium. The secretion of hydrophobins occurs at the tip of growing fungal hyphae [82,83], which was also observed for *T. viride* strain GZ1. Hydrophobins are deposited, mostly in the form of monomers, sometimes in the form of dimers and trimers [28,84], at the water–air interface, where they form a biofilm made of cross-linked self-assembled monolayers [85,86,87]. In our study, the presence of hydrophobin film on the surface of PLA and PET was confirmed by AFM analysis. Hydrophobin film usually has a structure of rodlets [88,89,90] similar to amyloid fibres. Both amyloids and hydrophobins have the structure of a β-sheet [91], and the β-sheet state is the final form of hydrophobin biofilm [88]. Using AFM, it was shown that hydrophobins from *Grifola frondosa* and *Pleurotus ostreatus* formed rodlets, with the length ranging from 50 to 105 nm, and the width ranging from 19 to 24 nm, usual for bilayer form [92,93,94]. It was shown that hydrophobin-producing fungi can stimulate enzymatic modifications to polymers, including poorly biodegradable PET. The in vitro fusion of bacterial cutinase and hydrophobin proteins derived from *Trichoderma* sp. was found to increase the level of PET hydrolysis. This increase was due to the fusion changing the conformity of the active cutinase enzyme site, resulting in better adhesion of the enzyme to the plastic [41]. Moreover, in a study by Takahashi et al. [95], cultivation of the industrial fungus *Aspergillus oryzae* in a liquid medium containing the biodegradable polyester polybutylene succinate coadipate (PBSA) led to high expression of the gene encoding class I hydrophobin RolA. Under these conditions *A. oryzae* produced also the cutinase CutL1. Using immunostaining, it was revealed that cutinase was specifically bound by RolA present on the surface of the polymer. In addition, the fusion of RolA with PETase significantly increased the level of PET hydrolysis, resulting in a weight loss of polymer of 26% in 4 days of incubations. The authors suggested that it was possibly due the change of the hydrophobic surface of PET into a hydrophilic surface, due to the presence of hydrophobins [42]. Based on the presented data we hypothesized that in the environment, *T. viride* is able to colonize pieces of plastics by forming a hydrophobin film on the surface of the polymer. The presence and the nature of this film is dependent on the chemical structure of the plastic waste. The role of hydrophobin in biodegradation is the improvement in effectiveness of hydrolytic enzymes produced by the fungus. Taken together, our results strongly indicate that *T. viride* GZ1 is a very promising candidate for the purpose of polymer biodegradation.

## 4. Materials and Methods

### 4.1. Polymer Materials

Commercial PET water bottles were washed, and after drying were cut into pieces (20 mm × 30 mm). The thickness of the obtained PET film equaled 0.152 mm. The PLA film was formed using a solvent-casting method. PLA 2002D type (NatureWorks^®^, Minnetonka, MN, US) was dissolved in chloroform. To obtain PLA film with the same thickness as PET film, 80 mL of prepared mixture (6% *w/v*) was poured onto glass Petri dishes and left for 2 days to enable the evaporation of the solvent.

### 4.2. Molecular Identification of T. viride GZ1 and Fungal Culture Conditions

The saprophytic fungus used in this study was isolated from the fruiting body of the *Cerioporus squamosus* (Huds.) Quél. The culture of the *T. viride* GZ1 fungus, stored in agar slopes at 4 °C, was transferred to a microbiological solid potato dextrose agar (PDA; Difco, Franklin Lakes, NJ, US) medium and incubated at 23 °C for 14 days. Fragments of the mycelium were transferred to the liquid potato dextrose broth (PDB; Biocrop, Warsaw, Poland) medium and incubated at 23 °C for 7 days. For the molecular identification of fungus, mycelia fragments were homogenized in liquid nitrogen. The genomic DNA was extracted using DNeasy Plant Mini Kit (Qiagen, Hilden, Germany) according to the manufacturer’s protocol. The purity and quantity of DNA were checked by electrophoresis on a 1.5% agarose gel with TAE buffer (40 mM Tris, 20 mM acetic acid, 1 mM EDTA) containing ethidium bromide, and by spectrophotometric measurement using the NanoDrop 1000 spectrophotometer (Thermo Scientific, Waltham, MA, USA). Identification of fungi was performed according to Raja et al. [96] and Vancov and Keen [97]. In the PCR reaction the following primers were used: ITS1_f (5′-CTTGGTCATTTAGAGGAAGTA-3′) and ITS2_r (5′-TCCTCCGCTTATTGATATGC-3′). The genomic region including internal transcribed region 1 (ITS1) (region between 18S rRNA gene and 5.8 rRNA gene), 5.8S rRNA gene, and ITS2 (region between 5.8S rRNA gene and 28S rRNA gene) was amplified. The PCR product was sequenced using the Sanger method (Genomed, Warsaw, Poland), and the screening for the homologous sequences from NCBI GeneBank was performed using BLASTN tool.

### 4.3. Characteristics of the T. viride GZ1 Strain

#### 4.3.1. Analysis of Fungal Metabolic Activity

The enzymatic activity of the microorganisms was determined based on their ability to synthesize hydrolytic enzymes, e.g., amylase, cellulase, lipase, and pectinase. Analysis of the hydrolytic activity of the fungus was performed according to Hrynkiewicz et al. [98] and Janczak et al. [20]. The enzymatic activity was expressed by W_act_ = S_h_^2^/(S_c_ × t), where S_h_—hydrolysis zone diameter; S_c_—colony diameter; and t—incubation time (168 h).

#### 4.3.2. Growth of *T. viride* GZ1 on Polymer Materials

The ability of the analyzed fungus to grow on a minimal solid medium containing sterile PLA and PET fragments was tested in accordance with the PN-EN ISO 846 standard. The polymer materials were sterilized by rinsing in 70% ethyl alcohol and then UV irradiation for 5 min in a UV Chamber (Bio-Rad, Munich, Germany). A spore suspension of *T. viride* strain GZ1 with a density of 10^6^ spores/mL was prepared according to the procedure described by Znajewska et al. [16]. The experiment in the liquid minimal medium was carried out in 200 mL flasks containing 5 pieces of PLA or PET film (30 mm × 30 mm) inoculated with 50 µL of spore suspension. As a control, pieces PLA and PET film were incubated in the liquid minimal medium. Flasks were incubated for 3 months in the dark at 23 °C. The experiments were performed in triplicate. The obtained pieces of PLA and PET polymers after 3-month incubation were used for all experiments (SEM, DCS, FTIR, viscosity measurements, Western blot, and AFM).

### 4.4. Assessment of the Properties of Polymeric Materials after Incubation with the Fungus

Before analysis, the fragments of PLA and PET polymers incubated in medium and in medium-*T. viride* were gently rinsed by submerging them several times in sterile MiliQ water.

#### 4.4.1. Scanning Electron Microscopy (SEM)

Small pieces of PLA and PET films before and after 3 months of incubation in liquid medium without the fungus (control) and in the presence of *T. viride* GZ1, were analyzed using a Quanta 3D FEG scanning electron microscope (Thermo Fisher Scientific, Walthman, MA, US). SEM was equipped with a field emission gun (Schottky FEG). The accelerating voltage range of the apparatus was from 200 V to 30 kV. Photographs of the topography of the samples were obtained using an SE detector (FEI). Before analysis, samples were covered with gold.

#### 4.4.2. Differential Scanning Calorimetry (DSC)

Changes in thermal properties of PLA and PET materials before and after the incubation with *T. viride* GZ1 were determined using a differential scanning calorimeter (Polymer Laboratories, Epsom, UK). Analyses were performed in a nitrogen atmosphere. The heating rate was 10 °C/min and the temperatures ranged between 25 and 180 °C for PLA and between 25 and 300 °C for PET material. Thermal parameters, i.e., glass transition temperature (T_g_), crystallization temperature (T_c_), heat of crystallization (ΔH_c_), melting temperature (T_m_), and heat of fusion (ΔH_m_) were established. The DSC analysis was performed in triplicate for all experimental variants.

#### 4.4.3. Fourier-Transform Infrared Spectroscopy (FTIR)

In order to evaluate the structural changes of PLA and PET polymers caused by *T. viride* GZ1, Fourier-transform infrared technique was applied. FTIR-ATR spectra were recorded on a Nicolet iS10 (Thermo Fisher Scientific, Waltham, MA, US) in the frequency range of 600–4000 cm^−1^, using germanium crystal. All spectra were scanned 64 times and recorded at a resolution of 4 cm^−1^.

#### 4.4.4. Viscosity Measurements

In order to analyze changes in the molecular mass of poly(ethylene terephthalate) and polylactide after *T. viride* treatment, viscosity measurements were performed. In the case of the PET samples, a solution consisting of phenol and 1,1,2,2-tetrachloroethane with a weight ratio of 60/40 was used. Chloroform was used to determine the PLA molecular weight mass. The viscosity measurements were performed employing the Ubbelohde type of capillary viscometer. A stopwatch was also used to determine the time flow of the analyzed solution. The time flow was measured at least four times at four different concentrations. The measurements were carried out at 25 °C.

### 4.5. Hydrophobins

#### 4.5.1. Detection of *T. viride* GZ1 Hydrophobins

Hydrophobin proteins were detected by Protein A immunoprecipitation and Western blot using a rabbit polyclonal anti-HFBI (hydrophobin I of *Trichoderma reesei*) antibody (Abcam, ab 225992). To 100 μL of 3-month old liquid fungi culture, 1 μL of anti-HFBI antibody was added. The mixture was incubated for 1 h at 0–4 °C with slow shaking. Next, 100 μL of Protein A immobilized on acrylic beads (Sigma Aldrich, Poznań, Poland) suspension was added to the incubation mixture. After incubation for 1 h at 0–4 °C with agitation, antigen–antibody–Protein A complexes were isolated by centrifugation and washed 3 times in 25 mM Tris-HCl buffer, pH 7.5. Antigen–antibody–Protein A complexes were dissociated by incubation with 25 mM glycine-HCl buffer, pH 2.5. The supernatant was neutralized with 25 mM Tris and subjected to SDS-PAGE and Western blot analysis. SDS-PAGE was performed according to the method described by Ogita and Markert [99]. Proteins were electrotransferred (90 V, 200 mA, 1 h, 0–4 °C) onto nitrocellulose membrane using a wet system (Bio-Rad, Munich, Germany). To visualize total protein, the membrane was stained with Ponceau S. After blocking in 5% (*w/v*) non-fat milk in TBS (Tris-buffered saline) (overnight, 0–4 °C), the membrane was incubated with primary antibody (polyclonal rabbit anti-HFBI (Abcam, Cambridge, UK) (dilution: 1:500 in 5% (*w/v*) non-fat milk with TBS) for 1 h at room temperature. After washing with TBS, the membrane was probed with a secondary antibody (goat anti-rabbit IgG conjugated to alkaline phosphatase; Sigma Aldrich, Poznań, Poland) (dilution: 1: 25,000 in TBS). The antigen–antibody complexes were visualized using NBT/BCIP Tablets (Roche, Penzberg, Germany).

#### 4.5.2. Detection of Hydrophobin Film—Atomic Force Microscopy (AFM)

In order to analyze surface topography and surface roughness, and to identify hydrophobin film, atomic force microscopy (AFM) (NanoScope MultiMode; Veeco Metrology, Inc., Santa Barbara, CA, US) was used. Analyses were performed in air, at ambient temperature, and in AFM tapping mode. To establish changes caused by hydrophobins on the surface of PLA and PET films the roughness parameters, i.e., the roughness average (R_a_), the root mean square (R_q_), and maximum roughness depth (R_max_) were determined.

### 4.6. Statistical Analysis

Statistical analyses were performed using PAST 3.10 software [100]. ANOVA (analysis of variance) followed by Dunn’s post-hoc test was used to determine significant differences between means. For the differences *p* < 0.05 was considered significant.

## 5. Conclusions

Fungi belonging to *Trichoderma* are able to colonize various polymers, both not biodegradable and biodegradable. In our study, the ability of *T. viride* strain GZ1 to produce hydrophobin proteins in the presence of polymer materials was confirmed, and the attachment of hyphae to the surface of polymers was shown. Moreover, the presence of hydrophobin film on the surface of PLA and PET films was demonstrated. The fungus produced hydrolytic enzymes including chitinase, pectinase, lipase, and protease. We could observe biodegradational changes of PLA and modifications of the surface of PET caused by the fungus.

We hypothesize that biodegradable changes of polymers caused by *T. viride* can be divided into stages:

1. The fungi adhere to the surface of the material and the expression of hydrophobin-coding genes increases.
2. Increase in the amount of hydrophobins in the environment of the fungus.
3. A hydrophobic film forms on the polymer surface.
4. Hydrolytic enzymes produced by *T. viride* are immobilized on the surface of the film and increase in effectiveness.
5. Biodegradable changes of PLA and PET.

Development of the processes of biodegradation of plastic using microorganisms is of great importance in the field of waste management. Among several proteins and metabolites produced by fungi that are involved in the degradation of polymers, hydrophobins are of crucial importance, since those proteins may be useful for the degradation of a wide range of polymers. Further field experiments with *T. viride* strain GZ1 might lead to the development of biopreparation to enhance the degradational processes of polymers.

## Figures and Tables

**Table 1:** DSC parameters of PLA and PET films before treatment, after 3-month incubation in medium, and after 3-month incubation in medium containing *T. viride* GZ1.

Sample	Tg [°C]	Tc [°C]	∆Hc [J/g]	Tm [°C]	∆Hm [J/g]
PLA	67.9	122.2	−18.4	157.4	18.1
PLA-medium	67.0	115.9	−28.8	157.8/151.2	30.5
PLA-T. viride	60.5	112.8	−32.0	157.8/149.2	36.1
PET	80.8	137.8	−26.8	248.9	37.5
PET-medium	77.9	138.0	−30.8	249.1	46.9
PET-T. viride	77.8	139.4	−31.6	249.1	47.8

T_g_, T_c_, T_m_—glass transition, cold crystallization, and melting temperatures. ΔH_c_, ΔH_m_—enthalpy of cold crystallization and melting processes.

**Table 2:** The values of intrinsic viscosity ([η]) and number average molecular weight (M_n_).

Sample	[η]	M_n_ (g/mol)
PLA	1.1645	52,919
PLA-medium	1.0948	47,044
PLA-T. viride	0.8678	36,765
PET	0.7270	32,226
PET-medium	0.7153	31,518
PET-T. viride	0.6983	30,496

**Table 3:** Values of roughness parameters of PLA and PET films before treatment, after 3-month incubation in medium, and after 3-month incubation in medium-*T. viride* GZ1. Values are the means from three independent measurements ±SD. The results were compared separately for each polymer and each parameter, and different letters indicate significant differences (*p* < 0.05).

Sample	R_a_ (nm)	R_q_ (nm)	R_max_ (nm)
PLA	2.89 ± 0.09 a	3.48 ± 0.08 a	37.0 ± 2.7 a
PLA-medium	3.78 ± 0.11 a	4.76 ± 0.07 a	47.0 ± 4.2 a
PLA-T. viride	8.19 ± 0.08 b	13.60 ± 0.12 b	157.0 ± 9.9 b
PET	2.12 ± 0.11 a	3.30 ± 0.09 a	53.6 ± 5.5 a
PET-medium	3.27 ± 0.20 a	4.19 ± 0.15 a	54.2 ± 8.1 a
PET-T. viride	7.67 ± 0.18 b	9.46 ± 0.11 b	73.2 ± 4.6 b

R_a_—roughness average, R_q_—root mean square, R_max_—maximum roughness depth.

## Data Availability

The data that support the findings of this study are available from the corresponding author upon reasonable request.
